# Genetic population subdivision of the blue swimming crab *(Portunus pelagicus)* across Indonesia inferred from mitochondrial DNA: Implication to sustainable fishery

**DOI:** 10.1371/journal.pone.0240951

**Published:** 2021-02-04

**Authors:** Hawis Madduppa, Rina Martaulina, Zairion Zairion, Resha Mukti Renjani, Mujizat Kawaroe, Nurlita Putri Anggraini, Beginer Subhan, Indri Verawati, Lalu M. Iqbal Sani

**Affiliations:** 1 Department of Marine Science and Technology, Faculty of Fisheries and Marine Sciences, Institut Pertanian Bogor (IPB University), Bogor, Indonesia; 2 Indonesian Blue Swimming Crab Association (Asosiasi Pengelolaan Rajungan Indonesia–APRI), Surabaya, Indonesia; 3 Center for Coastal and Marine Resources Studies, Institut Pertanian Bogor (IPB University), Bogor, Indonesia; 4 Oceanogen Environmental Biotechnology Laboklinikum, West Java, Indonesia; 5 Department of Aquatic Resources Management, Faculty of Fisheries and Marine Sciences, Institut Pertanian Bogor (IPB University), Bogor, Indonesia; National Cheng Kung University, TAIWAN

## Abstract

The blue swimming crab (BSC), *Portunus pelagicus* (Linnaeus 1758), inhabits coastal areas of Southeast and East Asia, and is one of high fisheries commodities with an export value for Indonesia and an increasing global market demand, annually. However, the data of genetic diversity and their spatial connectivity of populations in Indonesia are not yet known, even when it is important to inform stock unit management and sustainable use. This study aimed to determine the genetic diversity and differentiation of blue swimming crabs across Indonesian populations in different Fishery Management Area (FMA), and their spatial genetic connectivity, as well as to deliver implications for sustainable fishery. A total of 297 individuals were collected and amplified using cytochrome oxidase I mitochondrial DNA. This study has showed the highest values for haplotype and nucleotide diversity in the eastern part of Indonesia, where exploitation is relatively low. Significant genetic differentiation between populations (*F*_ST_ = 0.954; *p* < 0.001) and the fisheries management areas (*F*_ST_ = 0.964; *p* < 0.001) were revealed. Low spatial connectivity was observed between populations in a distance of at least more than 60 kilometers. This study suggests that BSC populations in Indonesia, likely have several stock units, and preferably different fisheries management plans and actions across the region thoroughly and simultaneously. This would be effective for management and their sustainable conservation.

## Introduction

The blue swimming crab (*Portunus pelagicus* Linnaeus, 1758) inhabits coastal waters of Southeast and East Asia [1]. The spatial connectivity of blue swimming crabs is influenced by their life cycle and other environmental parameters. Juveniles and adults of *P*. *pelagicus* inhabits benthic coastal environments for both estuaries and nearshore and females migrates into high salinity waters for spawning, but they seem not to return into the estuaries or nearshore waters after spawning [2], except the larval stage [3–5]. Juveniles of *P*. *pelagicus* couldn’t be found at nearshore waters where water salinity is less than 10 PSU [6], and this species would migrate from estuaries to marine waters as a reaction to lowered salinities [3, 7, 8]. The blue swimming crab has a life cycle with five larval stages, while larval duration might be for 26–45 days, afterwards follows the crab phase [4]. Since this species has moderately long planktonic larval stages and might have high mobility during the crab phase, it is occurs a high gene flow level within and between populations [9]

The Indonesian blue swimming crab fishery has developed rapidly since the 1990s and became an important source of coastal communities’ income. The crab meat has been exported approximately 20,000mt per annum over the last decade, primarily to USA markets. This product demands to be ecolabel certified and traceability systems now [10]. However, according to the government and industrial meat production, the average size of blue swimming crabs landed has been declining since 2008. The catch per trip is also fluctuated; even though the unit and dimension of fishing gears have increased. This trend also occurred in regions where the blue swimming crab fisheries developed earlier, and most of blue swimming crab fisheries tend to be overfishing. It’s seemed that the Indonesian blue swimming crab fishery trends are threatening the resources and economic sustainability. Therefore, Indonesia as one of the important supplier’s countries of raw crab materials for canning industry, which currently is the one commodity that is very important for more than 180 thousand woman pickers in miniplants and about 90 thousand fishermen in Indonesia, making it necessary to rescue the crab population from rapid decline or extinction, as has happened to other crab fishery (e.g *Callinectes sapidus* in Chesapeake Bay [11])

The genetic diversity reveals gene flow between and within the populations, and therefore could be used to determine the healthy of certain populations [12], and additionally determining the stability and resilience of populations [13]. Due to excessive fishing and environmental changes, many stocks have been depleted and some species might become endangered [14–16], and would be influencing the changes in population genetic diversity [17–19]. The decreasing of genetic diversity might occur in a long time before the actual effect is visible, which will have a long term affect of an increasing genetic drift and finally the loss of variability and adaptation ability [20, 21]. The genetic structure information of a population could effectively lead the fisheries management in a sustainable points of view [22–24].

The small size wild-caught proportion of *P*. *pelagicus* has been increasing in the last decades in Indonesia and suggests that this species is under over-exploitation. In dependency on production from capture, results can lead to the reduce the number of small crab populations. When the increasing rate in fishing effort is not comparable with the growth of the fish resources, fish stocks will be reduced and the result will be the decline in catches. This condition is known as biological overfishing [25]. Critical behavior of the crab is the development of life cycles that occurs in some places. The larval phase and spawning phase is in the open sea crab (off-shore), while the juvenile phase until the adults were are in coastal waters (near-shore) [4]. However, there have been a lack of information on genetic diversity and population subdivisions of *P*. *pelagicus* in Indonesia. Knowledge and identification result of reproductively isolated and/or genetically differentiated populations within a species are important for restocking programs [26, 27], and for the reconstruction of an appropriate management (i.e. the harvest strategy and harvest control rules) in this species [28]. Despite its high value, the population genetic structure of *P*. *pelagicus* in Indonesia has not been well studied unlike countries such as Malaysia, Australia and Thailand [29–31]. One study concerning morphometric character and mitochondrial 16S rRNA sequence of *P*. *pelagicus* has been conducted in Burru water, South Sulawesi as a small part of Indonesian waters [32]. Therefore, this study aimed to investigate the diversity and structure of genetics within and between populations of blue swimming crabs (*P*. *pelagicus*) across Indonesia, and to discuss the implications to blue swimming crab fishery sustainability.

## Materials and methods

### Tissue collection

The tissue sampling was conducted at fish landing sites around the harbor in each population during several expeditions in 2015–2017. Permits for tissue collection were not required, as the species is commercial and we were collecting samples at the enumerator sites of Indonesian Blue Swimming Crab Association (APRI). The tissue sample was collected by cutting a piece of small crab legs and stored in a 2 mL microtube containing 96% ethanol with a label containing the identity of the sample. A total of 297 tissue samples was collected from eleven populations representing six of Fishery Management Regions in Indonesia (Table 1).

**Table 1:** Number of samples (n), number of haplotype (*H*_n_), haplotype diversity (*H*_d_), and nucleotide diversity (π) from eleven populations representing six of fishery management area (FMA) across Indonesia, showing code for site shown in Fig 1.

Code	FMA	Site	Latitude	Longitude	n	Hn	Hd	π
BAT	571	Batu Bara	3.33058	99.50061	26	8	0.526	0.025
BLB	711	Bangka Belitung	-2.07287	106.1665	22	6	0.745	0.026
LAN	712	Pulau Lancang	-5.93320	106.5891	49	6	0.543	0.012
REM	712	Rembang	-6.69909	111.3393	47	5	0.723	0.016
PMK	712	Pamekasan	-7.22415	113.4465	60	12	0.542	0.015
LOM	713	Ekas, Lombok	-8.87284	116.4546	22	5	0.468	0.018
MAR	713	Maros	-5.04313	119.4764	18	6	0.621	0.020
BOM	714	Bombana	-4.83466	121.8036	14	4	0.396	0.008
PAM	714	Pamandati	-4.39428	122.5843	12	2	0.167	0.003
KEN	714	Kendari	-3.97911	122.5853	15	6	0.571	0.120
HAL	715	Halmahera	0.87616	127.4647	12	6	0.758	0.034

**Fig 1:**
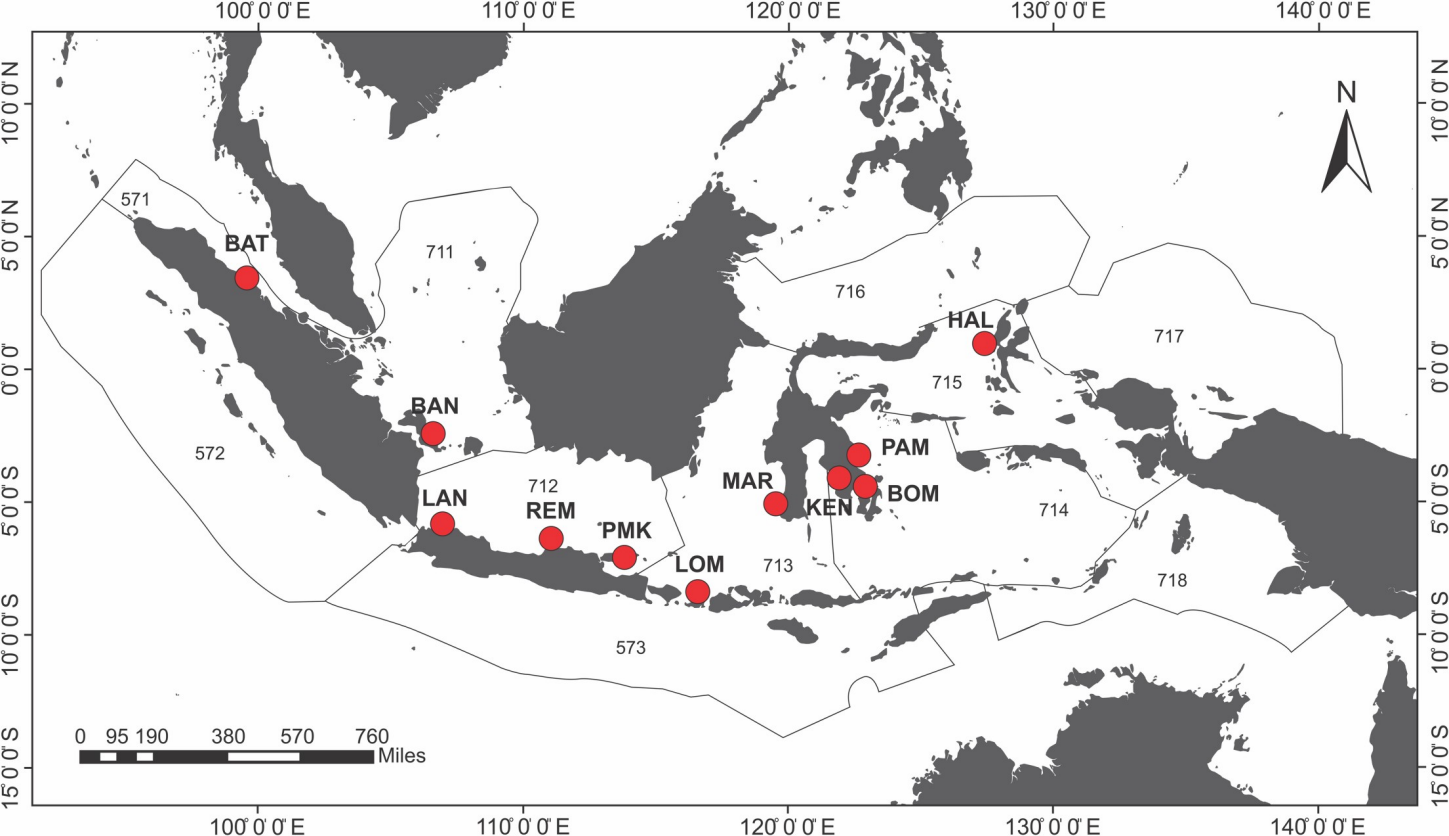
Tissue sample collection sites of blue swimming crab (*Portunus pelagicus*) at eleven populations (BAT: Batubara, BAN: Bangka Belitung, LAN: Pulau Lancang, REM: Rembang, PMK: Pamekasan, LOM: Ekas Lombok, MAR: Maros, BOM: Bombana, PAM: Pamandati, KEN: Kendari, HAL: Halmahera), representing six of fishery management areas (FMA) in Indonesia; FMA 571; 711; 712; 713; 714 and FMA 715.

### DNA extraction, amplification and sequencing

Extraction of genomic DNA for each sample was conducted by using extraction kit (Geneaid kit). A fragment of mitochondrial Cytochrome Oxidase subunit-I gene (COI) was amplified using the following primer set: CO1F 5’ AGA AGT GTA TAT TTT AAT TC -3’ and CO1R 5’-ATG TAG AAT ATC GAT AG-3’ [33]. Polymerase Chain Reaction (PCR) was conducted in 25 μl reaction volume containing 1–4 μl templates DNA, 2.5 μl of 10x PCR buffer (Applied Biosystems), 2.5 μL dNTP (8 mM), 2 μl MgCl_2_ (25 mM), 0.125 μl AmpliTaq Red™ (Applied Biosystems), 1.25 μl of each primer (10 mM), 1 μl 1x BSA, and 13.5 μl ddH_2_O. PCR conditions were: initial denaturation at 94°C for 15 s, followed by 40 cycles of denaturation at 95°C for 30s, annealing at 40°C for 30s, and extension at 72°C for 45s. The final extension step was conducted at 72°C for 10 min. The quality of PCR products was assessed by agarose gel electrophoresis and ethidium bromide staining and visualized using UV transilluminator. All good PCR products were sent to Sanger sequencing facility.

### Data analysis

Mt-DNA sequences were aligned and edited using Mega 6 [34]. The sequence dataset was submitted to BOLD under project entitled “Indonesian Crustaceans”, with Code: IDCRT (IDCRT001-20—IDCRT292-20), with the link as follow: http://www.boldsystems.org/index.php/MAS_Management_DataConsole?codes=IDCRT. GENETYX program was used to calculate genetic distance (D) among individuals in the intra population and inter populations. Based on Kimura 2-parameter model and 1,000 replication of bootstrap was constructed a Neighbour-Joining (NJ) tree in Mega 6 [35]. Analyzing a number of the haplotype (H) and haplotype diversity (Hd) by using DnaSP 5.10 [36], while nucleotide diversity (π) followed the method of Lynch and Creasef (1990) [37]. Subsequently, fixation index (*F*_ST_) is used to assess population differentiation [38] and determined by Arlequin 3.5 [39]. In order to investigate the phylogenetic relationship among haplotype, a minimum spanning tree was constructed in Network 4.6.1 (http://www.fluxusengineering.com). Then, Analysis Molecular Variance (AMOVA) was applied to assess genetic differentiation (*F*_ST_) among and within populations based on sites and Fishery Management Area (FMA) of blue swimming crab (*Portunus pelagicus*) across Indonesia [39, 40]. The *F*_ST_ will show whether between sites and the Fishery Management Area (FMA) is genetically different (different unit stock) or panmixing.

## Results

### Genetic diversity

A total of 40 haplotypes were observed in all populations, where highest was found in Pamekasan and the lowest in Pamandati (Table 1). The highest value of haplotype and nucleotide diversity was observed in Halmahera (0.758) and Kendari (0.120), respectively. Pamandati was observed to show the lowest for both haplotype (0.167) and nucleotide (0.003) diversity (Table 1).

### Population genetic structure

**Fig 2:**
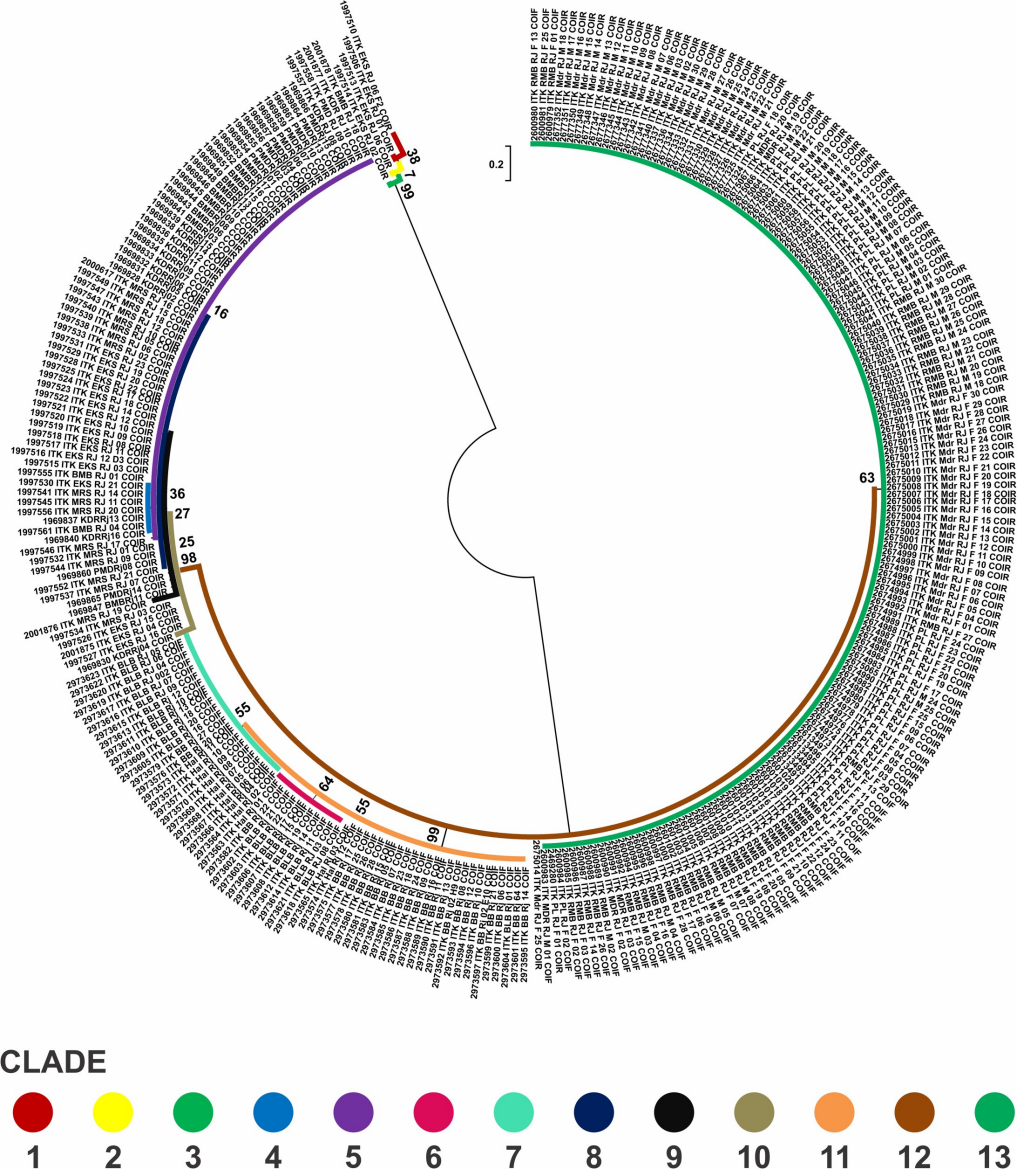
Reconstruction of phylogenetic trees blue swimming crab (*Portunus pelagicus*) markers CO1 using Neighbour Joining (NJ) Kimura 2 parameter models with 1000 bootstrap values of eleven populations across Indonesia using 297 sequences showing five clades: (1) Java Sea population (FMA 712), (2) Sulawesi and Lombok (FMA 713–714), (3) North Sumatera (FMA 571), (4) Bangka Belitung (FMA 711), and (5) Halmahera (FMA 715) from 13 haplotypes.

The reconstruction of phylogenetic trees *Portunus pelagicus* markers CO1 using Neighbour Joining (NJ) Kimura 2 parameter models with 1000 bootstrap values of eleven populations across Indonesia using 297 sequences showing five clades (Fig 2). The five clades as follows: (1) Java Island population (FMA 712), (2) Sulawesi dan Lombok (713–714), (3) North Sumatera (571), (4) Halmahera (715), and (5) Bangka Belitung (711).

The highest difference in genetic structure (*F*_ST_) among populations was shown between Lancang and Bombana of 0.9922, followed by populations between Kendari and Lancang (0.9860), and the closest populations between Kendari and Bombana (-0.0001) (Table 2).

**Table 2:** The genetic differentiation shown by Fixation Index (*F*_ST_) among sites of *Portunus pelagicus* populations across study sites in Indonesia.

Sites	Ekas	Maros	Lancang	Rembang	Pamekasan	Kendari	Bombana	Pamandati	Halmahera	Batu Bara
Maros	-0.0041									
Lancang	0.9373	0.9345								
Rembang	0.9353	0.9323	0.1494							
Pamekasan	0.9411	0.9388	-0.0119	0.1390						
Kendari	0.8236	0.8044	0.9860	0.9853	0.9860					
Bombana	0.8417	0.8238	0.9922	0.9917	0.9915	-0.0001				
Pamandati	0.8409	0.8229	0.9920	0.9915	0.9913	0.0075	-0.0775			
Halmahera	0.8812	0.8682	0.9749	0.9736	0.9748	0.9662	0.9815	0.9809		
Batu Bara	0.9084	0.9022	0.9731	0.9721	0.9731	0.9731	0.9822	0.9812	0.4585	
Bangka	0.9056	0.8984	0.97708	0.9760	0.9768	0.9748	0.9856	0.9852	0.0714	0.6424

The results of the analysis of AMOVA (Analysis of Molecular Variance) showed strong genetic subdivision among eleven populations (*F*_ST_ = 0.954, p<0.001) and Fishery Management Area (FMA) (*F*_ST_ = 0.964, p<0.001) (Tables 3 & 4). The highest difference in genetic structure (*F*_ST_) among Fisheries Management Area (FMA) was shown between 712 and 715 of 0.9785 whereas the difference in genetic structure (*F*_ST_) among populations in Fisheries Management Area (FMA) is 0.964 (Table 4).

**Table 3:** Fixation index (*F*_ST_) value of blue swimming crab (*Portunus pelagicus*) in Fisheries Management Area (FMA) of Indonesia.

FMA	571	711	712	713	714
711	0.6364				
712	0.9773	0.9793			
713	0.8825	0.8808	0.9479		
714	0.9135	0.9117	0.9639	0.7029	
715	0.4464	0.7140	0.9785	0.8602	0.8962

**Table 4:** Result of Analysis of Molecular Variance (AMOVA) and Fixation Index (*F*_ST_) based on sites and fishery management area (FMA) of blue swimming crab (*Portunus pelagicus*) across Indonesia.

Source of variation	d.f.	Variation (%)	*F*_ST_	Significance (*p*)
Sites				
Among populations	10	95.44	0.954	<0.001
Within populations	286	4.56		
FMA				
Among populations	5	96.36	0.964	<0.001
Within populations	291	3.64		

**Fig 3:**
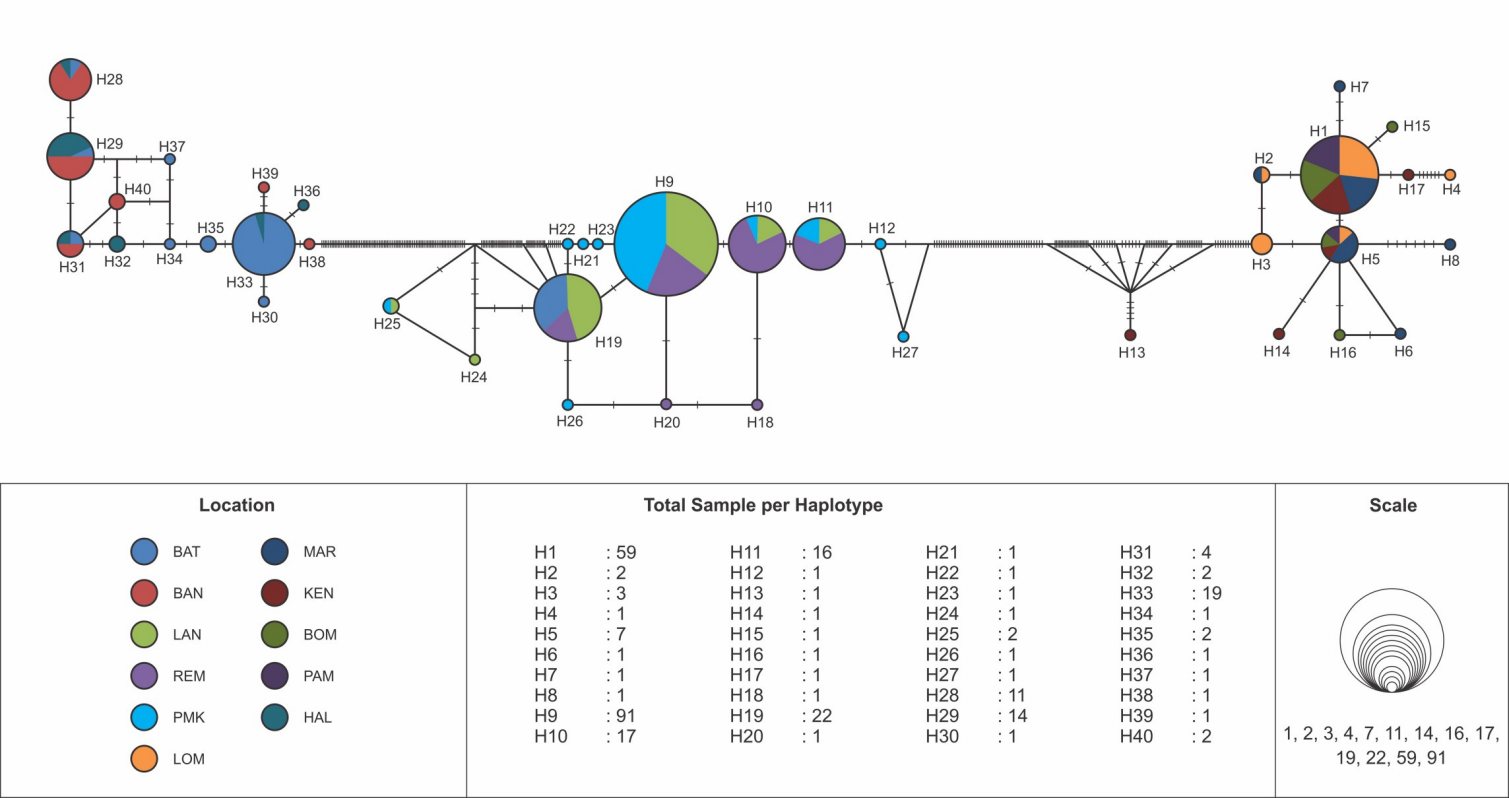
The haplotype network of blue swimming crab (*Portunus pelagicus*) using model TCS Network from a total of 297 sequences of eleven populations across sampling sites in Indonesia.

**Fig 4:**
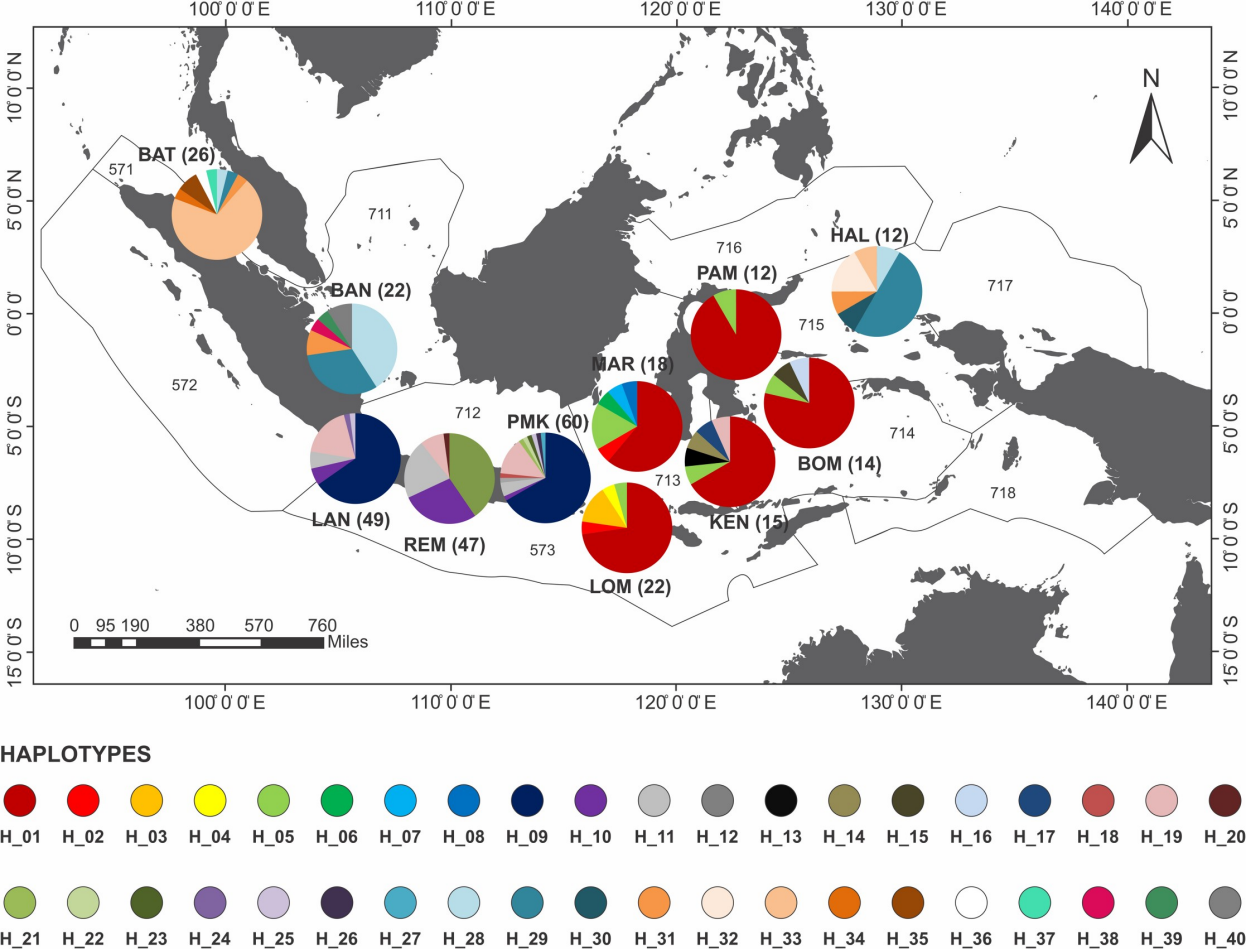
The haplotype distribution and composition of blue swimming crab (*Portunus pelagicus*) using model TCS Network from a total of 297 sequences of eleven populations across sampling sites in Indonesia. In bracket is number of sample per site.

**Fig 5:**
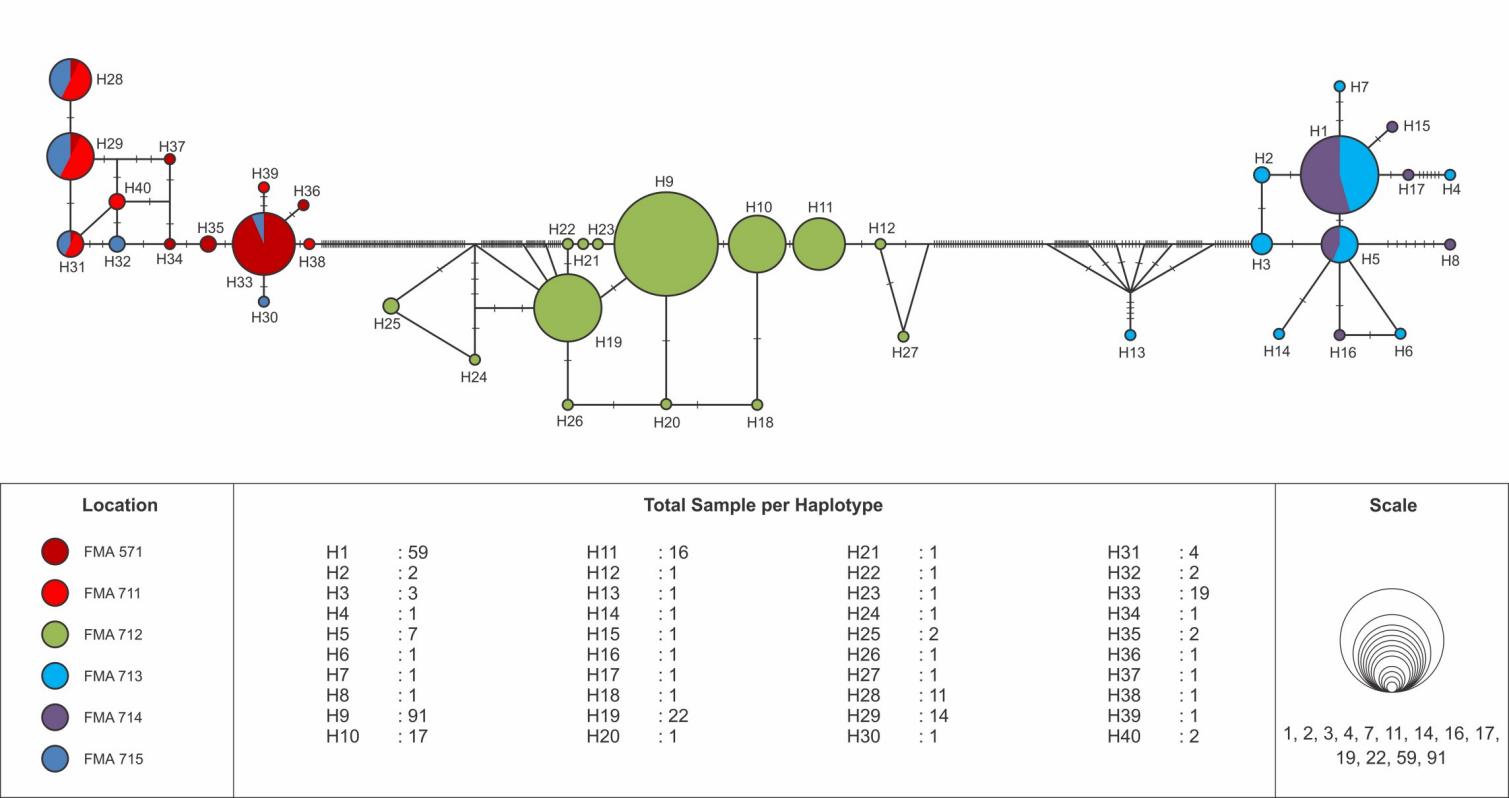
The haplotype network of blue swimming crab (*Portunus pelagicus*) using model TCS Network from a total of 297 sequences of eleven populations across Indonesia within fisheries management areas (FMA).

**Fig 6:**
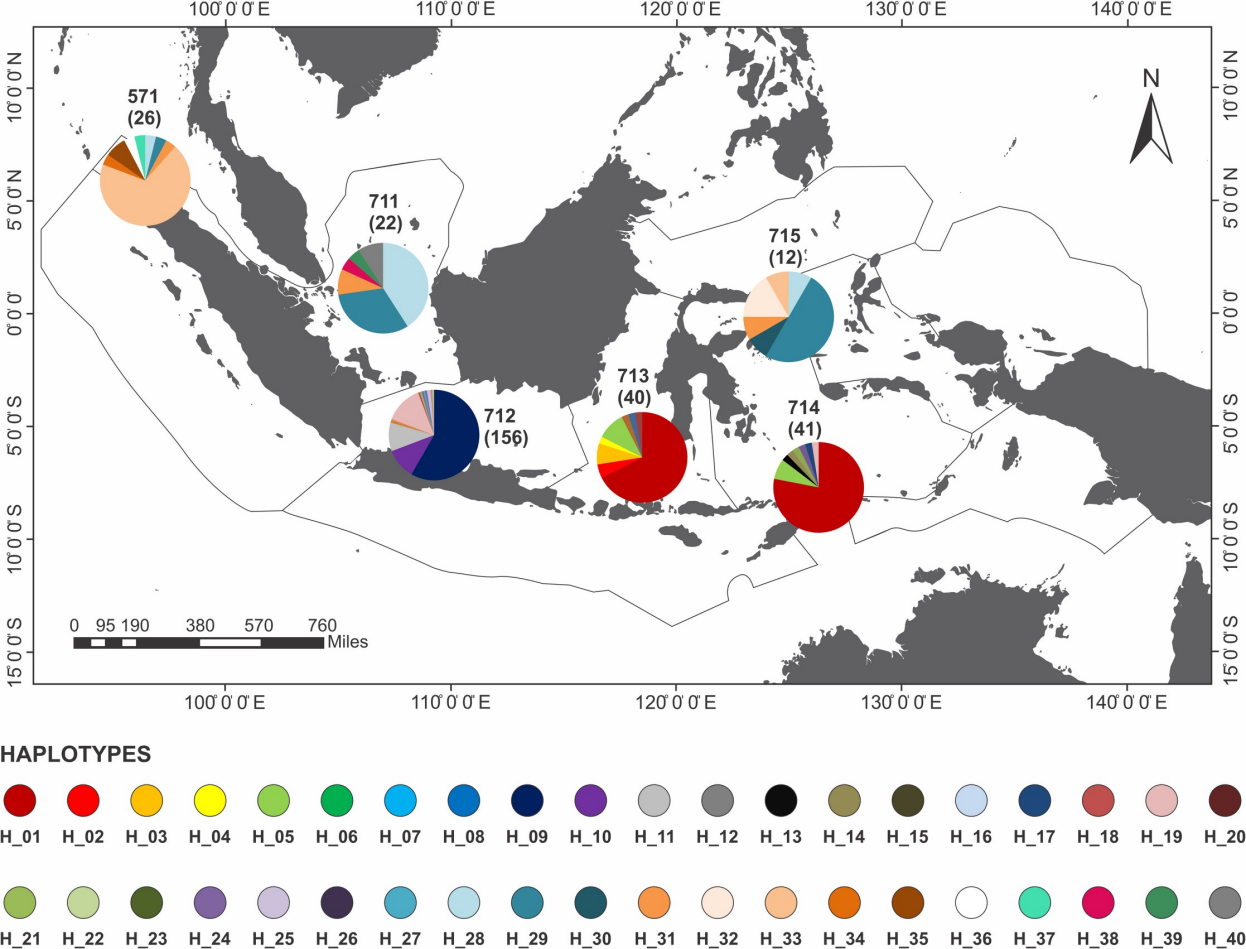
The haplotype distribution and composition of blue swimming crab (*Portunus pelagicus*) using model TCS Network from a total of 297 sequences of eleven populations across Indonesia within fisheries management areas (FMA). In bracket is number of sample per region.

The haplotype network of blue swimming crab (*Portunus pelagicus*) using model TCS Network from a total of 297 sequences of eleven populations across Indonesia showed that haplotype was distributed across Indonesia, but a significant difference FMA. The haplotype network, distribution and composition of blue swimming crab (*Portunus pelagicus*) using model TCS Network from a total of 297 sequences of eleven populations across sampling sites in Indonesia show that each site dominated by one haplotype (Figs 3 and 4). Figs 5 and 6 show that FMA 712 was separated differently to western Indonesia areas (FMA 571 and 711), and eastern Indonesia areas (FMA 713, 714, and 715).

## Discussion

### Genetic diversity

The amount of genetic diversity observed in the presented study sites may be due to fishing pressure regularly and crab are in a stage of self—environmental adaptation, with a population that is still available in nature, but are vulnerable to rapid decline or extinction. This case seemed to happen in the area of Southeast Sulawesi, where the fishing pressures is high, and also some areas in the north coast of Java, except Madura, where still a high genetic diversity can be found. The loss of genetic diversity has been experienced by other fisheries (e.g exploited anemonefish in Spermonde Archipelago, Indonesia [41]. The loss of genetic diversity will reduce the population ability to adapt to environmental changes [42]. Genetic diversity has a potential impact directly or indirectly on the population, and might follow the impact to the community and ecosystem [43]. Gen a living being is a fundamental reproductive unit in the formation of the individual, so the decline in genetic diversity will decrease the population for sustainable success. Moreover, population size should be estimated in determining the appropriate amount of catches [44].

The value of the population genetic diversity was affected by the number of haplotypes (Hn) that exist. This study has shown the highest value of haplotype and nucleotide diversity in the eastern part of Indonesia (e.g. Halmahera), might be due to least exploitation. A large number of haplotypes is directly proportional to the value of genetic diversity. The low genetic diversity of *P*. *pelagicus* population was also observed at the site of the Gulf of Saint Vincent, Spencer Gulf, and coastal areas of Western Australia [45]], because the number of haplotypes on each individual is dominated by a single haplotype. Meanwhile, on the east coast and the port of the western Australian waters high values were found for genetic diversity, because of the large number of different haplotypes. Similar results were observed in Thailand that showed a high haplotype diversity and low nucleotide diversity [46].

A total of 40 haplotypes were observed in all populations, where the highest was found in Pamekasan and the lowest in Pamandati. A diverse type of haplotype has an impact on the genetic diversity in a population. The more diverse types of composite haplotype population level of genetic diversity, genetic in the population will be higher and vice versa [47]. Genetic diversity was affected by the transfer of genetic material between populations of different locations [48]. Genetic diversity increases when there is a genetic input from other populations, called genetic migration. Great migration would lead to inbreeding and mixing of genes between different populations, in order to obtain variations in different genes [49]. The presence of a wide variety of genes of individuals in the population would be increasing the population ability in response to the changes of environmental conditions. High genetic diversity in the population of fish may protect may protect the crab from environmental interferences [50]. Taylor and Aarsen (1988) [51] explains that species with good adaptability will produce variations in phenotype and genotype both in response to particular environmental conditions so as to increase the ability of individuals to survive and proliferate.

### Population genetic structure

The reconstruction of phylogenetic trees for *Portunus pelagicus* on this study showing distinct five clades, of each Fisheries Management Area (FMA), except in Sulawesi and Lombok (713–714). Sezmiş (2004) [45] produced similar results, i.e., the formation of two major clades of *P*. *armatus* (previously known as *P*. *pelagicus*), each clade consisting of individual mixes of different populations in Australian waters. Phylogenetic interpreted as a model for representing approximately relationship ancestor organisms, molecular sequences or both [52]. This shows that although individual crabs are separated in different populations, a third of this population is closely related genetically or derived from a common ancestor. In addition, these results indicate that the crab migration patterns in the third phase of life of the population are still in the same location. Geographical distance is still within the scope of the population of the province, strengthening the case of the results obtained. Branching tree pattern matrix formed by the distance between pairs that can describe the population genetic fusion that occurs in the group [53].

A high value of *F*_ST_ was indicating strong subdivision and low gene flow between populations. The current study is similar to some of previous studies, which have revealed sub structure on the *P*. *pelagicus* populations. In Australia, some studies showed Australian waters are divided into four subpopulations based on different markers (i.e. allozyme polymorphism, COI and microsatellite). The study of Bryars and Adams [28] using allozyme showed subpopulations between populations (such as West Coast, Spencer Gulf, and Gulf St. Vincent in South Australia and Darwin-Gove in the Northern Territory), and within each subpopulation among the sites. Other population genetic structure of *P*. *pelagicus* was investigated from 16 different assemblages throughout the geographic range of this species in Australian waters [45]. This investigation used six microsatellite loci and a 342 bp fragment of cytochrome oxidase subunit I (COI), and found significant genetic heterogeneity.

The current study is contradictory as previously assumed by Yap *et al*. [29] that the *P*. *pelagicus* who has an extended planktonic larval stages would potentially has high larval dispersal and might occur of extensive gene flow between conspecific samples within geographic mesoscale that is from ten to hundreds of kilometres. In contrast with the observed result in China, population genetic structure has a limited occurrence with a high level of gene flow along the distribution areas of this species in China [46]. While in Malaysia, microsatellites analyses indicated low levels of genetic differentiation among the *P*. *pelagicus* populations [31]. This result also supports that *P*. *pelagicus* are capable of moving substantial distances, with one recorded as traveling 20 km in one day in Moreton Bay, Queensland [54, 55]. Other observation concerning *P*. *pelagicus* is also regarded as a potential species vagile since adults are able to travel in geographic mesoscale daily [4], which showed by the current study a mixed population within a region. It seems that within a mesoscale regions, blue swimming crab population is tend to be mixed as also showed by the current study (e.g. within Java Sea region), but will be different genetically in great scale.

### Implications to fishery management

The blue swimming crab has a high productivity and rapid growth rates [56], thus depleted blue swimming crab stocks could recover quickly by restoring and maintaining breeding sized crabs in the stock. The strong subdivision and low gene flow between populations as shown by a high value of *F*_ST_, suggest that each fisheries management area (FMA) in Indonesia should be treated differently by developing fishery management plan and strategic action at each management region. The biological characteristics of blue swimming crab, the coherent organized nature of the industry, and its reliance on sustainability conscious export markets makes the blue swimming crab fishery strategic for starting the process and inclusive collaborative management model for coastal fisheries [57]. Information on the genetic diversity of this crab can be further used as a database on mapping potential areas of Indonesia blue swimming crab. Crab management in each population needs to be done thoroughly and simultaneously. It is required in the implications for sustainable crab fishery, because it can determine an effective strategy that is continued. This study provides insights into population genetic structure of *P*. *pelagicus* in Indonesia, including conservation genetics, and utilization management of this species on a sustainable basis.
